# Impact of Rising Temperature in the Deposition Patterns of Bioactive Compounds in Field Grown Food Barley Grains

**DOI:** 10.3390/plants10030598

**Published:** 2021-03-22

**Authors:** Mariona Martínez-Subirà, Marian Moralejo, Eva Puig, María-Paz Romero, Roxana Savin, Ignacio Romagosa

**Affiliations:** AGROTECNIO-CERCA Center, University of Lleida, Av. Rovira Roure 191, 25198 Lleida, Spain; mariona.martinez@udl.cat (M.M.-S.); marian.moralejo@udl.cat (M.M.); eva.puig@udl.cat (E.P.); mariapaz.romero@udl.cat (M.-P.R.); roxana.savin@udl.cat (R.S.)

**Keywords:** *Hordeum vulgare*, grain filling, heat stress, β-glucans, arabinoxylans, phenolic compounds, antioxidant capacity

## Abstract

High temperatures at the end of the season are frequent under Mediterranean conditions, affecting final grain quality. This study determined the deposition patterns throughout grain filling of dry matter, dietary fiber, phenolic compounds and antioxidant capacity for four barley genotypes under two contrasting temperatures. Deposition pattern for dietary fiber followed that of grain weight. Genotypic differences for duration were more significant than for rate. Anthocyanins followed a second-degree polynomial pattern, reaching a maximum before grain maturation. Free and bound phenols decreased as grain developed, suggesting that they are synthesized in early stages. Rate of bound phenols deposition was more sensitive to genotypic changes. Overall, antioxidant capacity decreased over time; the decay being less steep under stress for all genotypes. Heat stress negatively affected grain weight. It did not alter the profile of β-glucans and arabinoxylans deposition but positively changed the accumulation of some phenolic compounds, increasing the antioxidant capacity differentially across genotypes. These results support the growing of food barley in high-temperature stress-prone areas, as some bioactive compound and antioxidant capacity will increase, regardless of the smaller grain size. Moreover, if a market develops for food-barley ingredients, early harvesting of non-mature grain to maximize antioxidant capacity should be considered.

## 1. Introduction

Barley (*Hordeum vulgare* L.) grain contains a variable amount of bioactive compounds with known health-promoting properties, such as dietary fiber (β-glucans, arabinoxylans, cellulose, lignin, and lignans), phenolic compounds, tocols, sterols and folates [1]. β-glucans are major non-starch polysaccharides present in cell walls. They are linked to the maintenance of normal blood cholesterol levels [2] and the reduction of blood glucose after meals [3], as well as improving the responsiveness of the immune system to infectious diseases, inflammation and some types of cancer [4]. Arabinoxylans constitute a fraction of dietary fiber, which have positive effects on the human digestive system [5]. Phenolic compounds are a large class of secondary plant metabolites, which can be found free or bound to compounds in the cell wall of the barley grain. Phenolic compounds, with their strong antioxidant power, are associated with the reduction of cardiovascular disease, inflammation and a diversity of cancers [6]. Interest in the health benefits of barley has led to an increased focus on these bioactive compounds in mature grain [7,8].

The effective grain filling phase (between the end of the lag phase and physiological maturity) is when dry matter and bioactive compounds accumulate, determining the final weight and nutrient composition and the quality of the grain [9]. It is well documented that the accumulation of dry matter during grain filling follows a sigmoid pattern [10]. This is characterized by three phases: lag phase (fertilization and rapid cell division), the effective grain filling period (accumulation of reserve components) and the maturation drying phase (loss of water content and reaching “physiological maturity”, i.e., maximum dry matter accumulation) [11,12]. In contrast, less is known about the accumulation patterns of the bioactive compounds during barley grain filling. Changes in non-starch polysaccharide accumulation during cereal grain development have been observed. It has been reported that β-glucans increase linearly, starting during endosperm development and continuing until ripening in barley grain [13]. In wheat, which is often used as a model similar to barley, the β-glucan concentration initially increases and then decreases slowly throughout development to a low concentration at maturity [14]. Arabinoxylan was reported to appear in barley during early cellularization, changing its structures during endosperm development from a highly substituted form to a less substituted form [15]. Similar to barley, the distribution pattern of arabinoxylans deposition in wheat shows a rapid increase in concentration at the end of the cell division and expansion phase, until it reaches the maturation phase where it remains constant [14]. Different patterns of variation in phenolic compound content have also been observed during grain filling. For instance, in wheat, total bound phenolics peak in the early stage of development [16], while the anthocyanin concentration first increases sharply during the immature phase and then decreases from 25–33 days after anthesis and onward [17].

The Intergovernmental Panel on Climate Change (IPCC) has projected that the global warming trend from 1986–2005 to 2081–2100 will show a temperature increase of 0.3 °C to 1.7 °C [18]. Therefore, current concerns exist on the impact of global climate change on the production of crops such as barley [19,20,21]. Future growing conditions will expose plants to variable and extreme climate change factors, impacting global agriculture, so future research in this area is essential [21] to take adequate adaptation measures. High maximum temperature during the grain filling period is one of the most relevant abiotic stresses under Mediterranean conditions. In fact, it is expected to be more frequent due to climate change [22]. The clear effects of high temperature on the reduction of grain weight are well documented in the literature [23]. However, high temperatures can also induce various physiological, biochemical and molecular responses in plants. In a recent study, we observed that the thermal stress during grain filling affects the final grain weight and changes the relative composition of β-glucans, arabinoxylans and more than 50 phenolic compounds in the mature grain [24]. Therefore, understanding the dynamics of the accumulation of these bioactive compounds under heat stress is another crucial aspect for enhancing the nutritional value of the barley grain at harvest. Insights into the time course and compositional changes of bioactive compounds during grain development is an important aspect for improving the nutritional quality of barley. Hence, in the present study, we compared the pattern of accumulation of dry matter and bioactive compounds (β-glucans, arabinoxylans, total free and bound phenolic compounds and their antioxidant capacity) in four barley genotypes exposed to continuous high temperature under field conditions. Identifying the timeframe when the maximum content of these bioactive compounds may occur could be useful for agronomic practices and also for promoting further research into whether non-mature barley grain could be used as a functional ingredient in the elaboration of healthy cereal-based food products. Therefore, the main aims of this study were (i) to determine the deposition pattern and antioxidant capacity throughout grain filling and (ii) to analyze the effect of high temperature on the accumulation of these components. This research could provide further knowledge about the deposition patterns of different bioactive compounds in barley under two contrasting temperatures in field conditions since the few published works in barley have focused on changes in deposition patterns of individual bioactive compounds under stress-free conditions [13,15]. To the best of our knowledge, this is the first time that a study has explored the deposition patterns of the main health-promoting components in food barley genotypes growing in field conditions under two contrasting temperatures.

## 2. Results

### 2.1. Grain Weight

**Figure 1:**
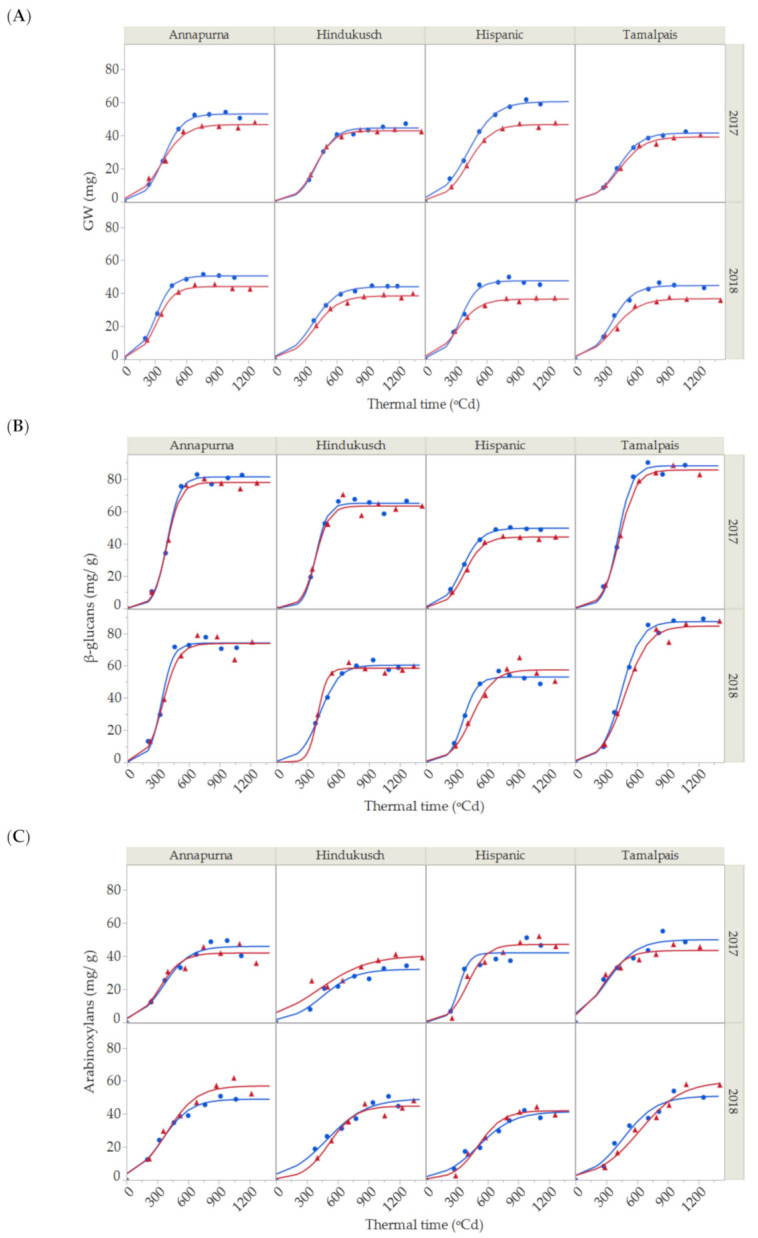
(**A**) Dynamics of grain weight growth, (**B**) β-glucans and (**C**) arabinoxylan contents for the two seasons (2017 and 2018) and four barley genotypes under control (blue points) and thermal stress conditions (red triangles). Solid lines represent the 3-parameter logistic fit for control (blue) and stressed conditions (red) for grain weight, β-glucans and arabinoxylan (Total *R*^2^ = 99.07%, *R*^2^ = 98.30% and *R*^2^ = 94.54%, respectively).

As expected, the standard 3-parameter logistic growth was found to be the most appropriate model for describing the grain filling process for grain weight (GW) in the 16 genotypes × year × environment combinations (Figure 1A). The overall *R*^2^ value for the fitting of the 16 logistic curves to the GW data was ca. 99%. Table 1 shows the partitioning of variability for maximum grain weight, growth rate and duration for the 16 standard logistic curves in Figure 1A.

A significant environmental effect was detected for maximum grain weight, which was quantitatively more important than the genotypic effects and the other terms in the model. In the control treatment, average maximum grain weight was highest for Hispanic (54.0 ± 6.4 mg), followed by Annapurna (51.7 ± 1.2 mg), Hindukusch (44.2 ± 0.3 mg) and Tamalpais (43.1 ± 1.6 mg) (Figure 1A). The maximum grain weight of the heat stressed plants were lower than in the control plants (7–22% average decrease). Although not significant, the reduction in maximum grain weight caused by high temperature seemed to be more related to the rate (*p* = 0.1207) than the duration (*p* = 0.9669) of the grain filling and a small, non-significant reduction in the grain filling rate was most often observed under heat stress. Significant variations in the duration of grain weight among genotypes were observed during grain filling (Table 1). On average, the duration of the period was shorter for Annapurna compared with the other genotypes. A lack of statistical significance for the genotype × environment interaction for any of the three parameters (growth rate, duration and maximum) (Table 1) was surprising, as we could infer from Figure 1 that some genotypes seemed to be more affected by high temperatures than others, i.e., Hispanic vs. Hindukusch. This could be related to the model structure in which interactions involving the year were pooled into the error term. This was a consequence of the absence of full replications, resulting in poor detection power.

### 2.2. β-Glucan and Arabinoxylan Contents

β-glucan concentration steadily rose at the beginning of the grain filling period and then accumulated substantially between the 600 to 800 growing degree-days after anthesis (GDA; Figure 1B), reaching a maximum peak. Thereafter, the β-glucan content did not change substantially. The maximum, rate growth and duration for β-glucans during grain filling were estimated from fitting the logistic curve and analyzing the variance for the estimates of β-glucans, as shown in Table 2.

In this study, the only significant effect associated with the accumulation of β-glucans was the genotype. Tamalpais and Annapurna, known as high β-glucan content genotypes, had maximum β-glucan concentration values of 87.8 ± 0.4 mg/g and 77.6 ± 3.6 mg/g, respectively, followed by Hindukusch (62.7 ± 2.3 mg/g) and Hispanic (51.3 ± 1.7 mg/g). Genotypic variations were also found for the duration of β-Glucan accumulation. Again, on average, Annapurna needed less thermal time to increase the content of β-glucans from 5% to 95% of the final value during grain filling. No significant environment or genotype × environment interaction was detected (Table 2).

During grain filling, the arabinoxylan content also increased rapidly from shortly after the end of anthesis (200 GDA) up to the maturation and desiccation phase (800 GDA) (Figure 1C). Arabinoxylans were fit by a logistic curve with an overall *R*^2^ of 94.54%. Partitioning of the total variability of arabinoxylans is shown in Table 3, with the maximum content, growth rate and duration for the 16 logistic curves shown in Figure 1C. Neither the environment nor the main genotype main effects showed statistical differences for any of the three parameters. The year—that is, the uncontrolled environmental differences associated with the growing season—was the only significant main effect and only for duration.

**Figure 2:**
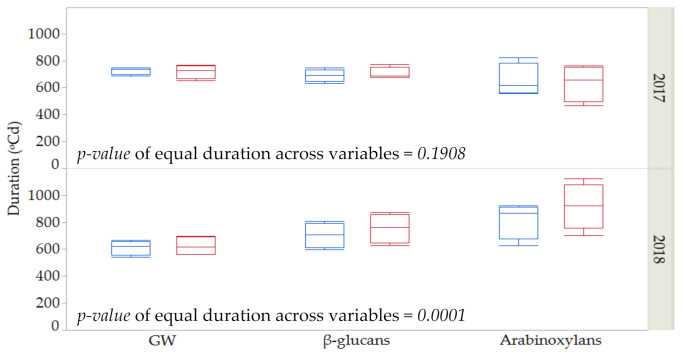
Estimates and standard error for duration for grain weight (GW), β-glucan and arabinoxylan contents across years (2017 and 2018) under control (**blue**) and stressed conditions (**red**) among four genotypes.

As shown in Table 1, Table 2 and Table 3, durations of grain weight and fiber deposition were, in general, more affected by year than by genotype or by the imposed thermal stress. Figure 2 shows the average duration for these three variables across years and control vs. stress conditions for the four genotypes. No differences in the duration of the three variables were found in the first year (*p* = 0.1908, ANOVA table not shown). However, significant differences were found among the three variables for the second season (*p* = 0.0001, ANOVA table not shown). In 2018, a more favorable year in terms of meteorological conditions during grain filling, deposition of arabinoxylans took longer than for GW and β-glucans under stress and control conditions and for the four genotypes.

### 2.3. Anthocyanin Contents

**Figure 3:**
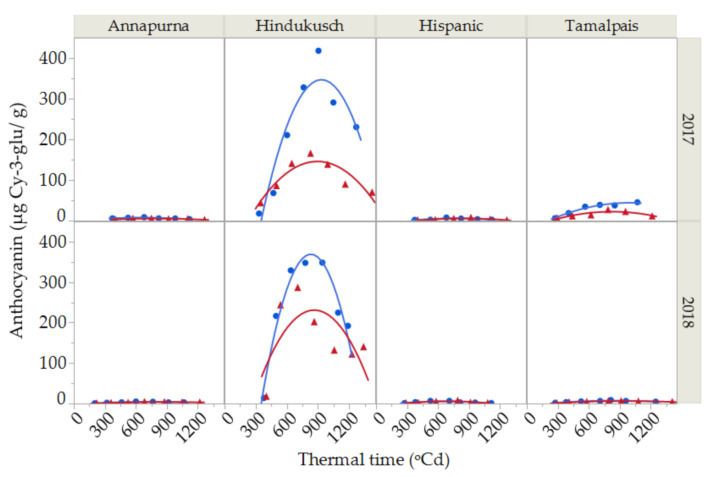
Anthocyanin content during grain filling for two seasons (2017 and 2018) and for the purple barley genotype (Hindukusch) under control conditions (blue circles) and thermal stress (red triangles). Solid lines represent the best fit (second-degree polynomial curve, Total *R*^2^ = 86.45%) for control (**blue**) and stressed (**red**) conditions, respectively.

Although anthocyanins were recorded during the course of grain filling for all four genotypes (Figure 3) in this study, the dynamics of anthocyanin deposition were only studied in Hindukusch, the only purple genotype, as the other three non-colored genotypes showed irrelevant extremely low values. The anthocyanin content in Hindukusch during the course of grain filling were best described by a second-degree curve (Figure 3). The synthesis of anthocyanins started relatively late in grain filling (at about 400 GDA), reaching a maximum peak in maturity (800 GDA) and decreasing throughout grain desiccation. The average maximum content during both growing seasons was 383 ± 49 µg Cy-3-glu/g in the control and 226 ± 85 µg Cy-3-glu/g in the heat treatment. Thus, the reduction of anthocyanin content between the maximum values reached at maturity until harvest time was in the order of 40% (Figure 3).

The anthocyanin content of barley grain grown under plastic cover was significantly lower than in the controls (Figure 3, Table 4). Furthermore, the significant environment × thermal time interaction suggested that the rate of deposition of anthocyanins changes under control and stress conditions (Table 4).

### 2.4. Free and Bound Phenol Contents

**Figure 4:**
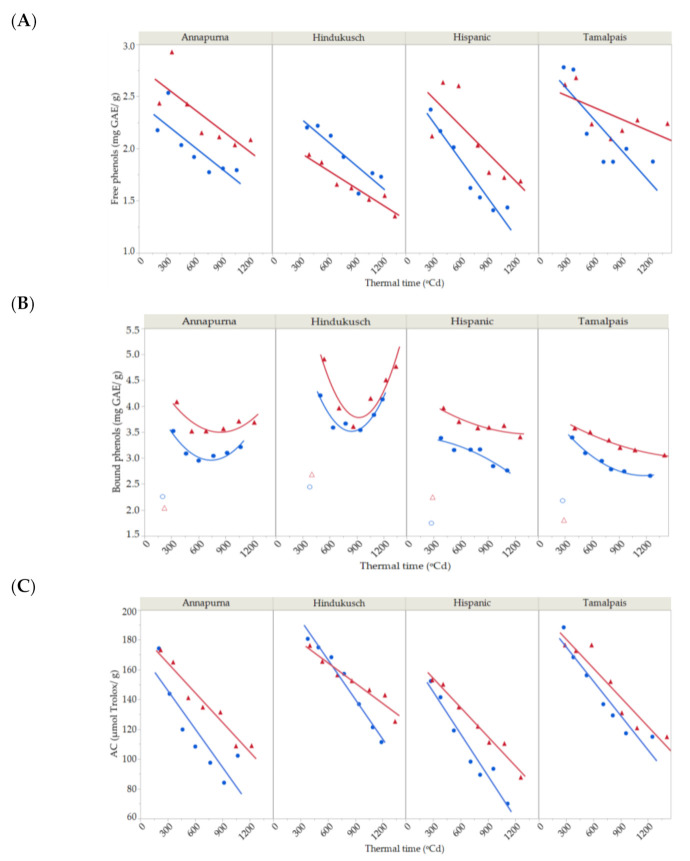
Dynamics of free (**A**), bound (**B**) phenolic compound content and (**C**) antioxidant capacity during grain filling for four barley genotypes in 2018 under control (blue circles) and thermal stress conditions (red triangles). Solid lines represent the best fit (first- and second-degree polynomial curve for free and bound phenolic compounds and first-degree for antioxidant capacity. Total *R*^2^ = 81.19% and *R*^2^ = 96.65%, *R*^2^ = 93.45%, respectively).

Free and bound phenols were determined for the four genotypes under normal and stress conditions in the course of grain filling in the second year. Free phenols continuously decreased throughout the grain filling period (Figure 4A) by around 30% between the maximum content at the beginning of grain filling until harvest time. Their dynamics were best fitted by a simple first-degree linear model, which was used for partitioning total variability (Table 5). The bound phenol content was more variable, fluctuating from one genotype to another. The bound phenols content decreased for Hispanic and Tamalpais with time (Figure 4B). However, Hindukusch and Annapurna followed a different pattern. Initially, the bound phenolic decrease occurred similarly to the other genotypes, but the drop rate reduced after 700 GDA and remained relatively constant or increased significantly in the case of Hindukusch. High temperature exposure did not alter these patterns of accumulation of bound phenols in all genotypes.

Both genotype and environment—that is, control vs. imposed heat stress, main effects and their interaction—were significant for free phenols (Table 5). Overall, the genotype seemed to be more important than the environment. Under heat stress, concentrations were higher for the three non-colored genotypes (7–16% average increase during grain filling) but not for Hindukusch, which had more free phenols in the control. The lack of significant interactions with thermal time suggested a common dynamic of free phenol content across genotypes, control vs. imposed stress. Bound phenol concentrations were significantly affected by genotype and environment main effects, but no genotype × environment interaction was detected. The significant genotype × thermal time interaction for bound phenols suggested that the rate of deposition of these compounds changes with genotype, as seen in Figure 4B. Hindukusch clearly behaves differently than the others.

In the control treatment, the maximum content of free phenols in immature grain was observed in Tamalpais (2.8 ± 0.1 mg GAE/g), while the highest levels of bound phenols were seen in Hindukusch (4.2 ± 0.1 mg GAE/g). These two genotypes maintained their higher contents compared to the other genotypes once the grain was ripe, but the free phenol content decreased by approximately 30% for Tamalpais and the bound phenol content decreased by approximately 3% for Hindukusch at the end of the grain filling.

### 2.5. Antioxidant Capacity

Antioxidant capacity sharply decreased as dry matter increased during grain filling (Figure 4C). The antioxidant capacity ranged from 152 ± 7 to 188 ± 9 µmol Trolox/g in the first measurements and from 70 ± 1 to 115 ± 3 µmol Trolox/g at the end of the experiment, a decrease of around 40% during grain growth. The antioxidant capacity was modelled by a simple first-degree model, which explained 95.36% of the total variability. Partitioning of the total variability for antioxidant capacity based on this linear trend is shown in Table 5. Genotype, environment and its interaction were highly significant. Tamalpais and Hindukusch had the highest antioxidant capacity values both in early immature grain (188 ± 9 and 181 ± 5 µmol Trolox/g) and mature grain (115 ± 3 and 111 ± 2 µmol Trolox/g). This was expected as these two genotypes had the highest content of free and bound phenols. The antioxidant capacity was higher for genotypes grown under heat stress (13%), except for Hindukusch. The highest increase in the antioxidant capacity due to heat stress was observed in Annapurna and Hispanic (on average 26% and 13%, respectively), the two genotypes that also had a greater increase in free (16% and 18%, respectively) and bound phenols (16% and 12%, respectively) due to the imposition of thermal stress. The thermal time × environment interaction was also significant, suggesting that the decay observed in the control plots was steeper than for the induced-stressed ones (Figure 4C).

## 3. Discussion

Barley (*Hordeum vulgare* L.) grains are rich in bioactive compounds with health-promoting properties, which are genetically and environmentally regulated. The four different barley genotypes selected for this study widely differed in an array of bioactive compounds, potentially susceptible to heat stress from 15 days after heading to physiological maturity.

As discussed in previous studies [24,25], the use of polyethylene film chambers to increase the maximum temperature under field conditions resulted in reduced incident radiation (up to 15% at noon on very sunny days). The relationship between direct and diffuse incoming radiation was changed, favoring the latter. Then, the reduction in incoming radiation was compensated by increasing the radiation use efficiency, and the source-balance hardly changed during grain filling [25]. Therefore, the main effect on grain weight was due to the increase in maximum temperatures.

The duration of grain filling was not affected by heat stress when measured on growing degree-days, as shown by others [26]. The differences in final grain weight between the controls and heat conditions might be due to the differences in the rate of the grain filling period since a positive correlation was found between growth rate and the effect of high temperatures on the maximum weight. In fact, some authors have suggested the selection of genotypes with higher filling rates as the best strategy for increasing grain weight [26]. Then, the weight reduction by thermal stress exposure was probably more related to a direct effect on the grain growth capacity, as proposed by MacLeod and Duffus [27]. High temperatures may induce inactivation of sucrose synthase, reducing the starch synthesis, reflected in the reduction of grain weight.

Barley is an important source of dietary fiber (β-glucans and arabinoxylans). No apparent dilution effect of these carbohydrates was detected with grain growth. The accumulation of β-glucans (expressed in mg/g of dry matter) through grain filling also followed a sigmoid pattern, very close to that of grain weight, suggesting that β-glucans were synthesized all through grain filling at the same rate as dry matter. The arabinoxylan content also followed a sigmoid pattern, although the fit to the 3-parameter logistic model was not as good as that for grain weight and β-glucans. According to De Arcangelis et al. [13], β-glucans increased linearly from endosperm development to maturation, whereas, seemingly contrary to our findings, Wilson et al. [15] reported that arabinoxylans accumulated significantly during early cellularization and changed their structures during grain development. They observed that the xylan backbone is heavily substituted with arabinose residues during early grain development. The apparent contradiction between early synthesis and our continuous deposition could be related to the analytical method used to quantify arabinoxylans. The standard D-xylose enzymatic procedure may not be fully adequate to quantify arabinoxylan levels during the early stages of grain filling, where the polysaccharide is in a highly substituted form with arabinose side chains. Future studies would be necessary to determine whether greater quantification of arabinoxylan can be obtained by including a pre-treatment with debranching enzymes such as arabinofuranosidase in the early stages of grain filling.

In this study, genotypic differences were more important than environmental effects on the β-glucan and arabinoxylan contents. Although we expected more significant differences associated with environmental factors, as reported by Swanston et al. [28], our results suggest that the content of β-glucans in barley was determined mainly by the genotype. This is in line with Molina-Cano et al., who also considered that the genetic effects were more important than the environmental conditions in the final β-glucan content [29]. Despite the few studies that have examined the variation in arabinoxylan levels due to heat stress, it has been shown that the heat effect increased the total arabinoxylan content in wheat [30]. However, we have previously observed that the final arabinoxylan content in barley was actually caused by an indirect concentration effect of the same amount of arabinoxylans in lighter grains and not by an apparent direct response to the heat stress imposed [24].

In general, no statistical differences were found for the duration of dry weight, β-glucan and arabinoxylan deposition during grain filling, suggesting that the maximum content for dietary fiber roughly coincides with maximum dry weight, that is, at or near physiological maturity. However, in 2018, a more favorable year in term of meteorological conditions during grain filling, the deposition of arabinoxylans took longer than for GW and β-glucans under stress and control conditions and for the four genotypes, suggesting that the thermal time of analyzable arabinoxylan accumulation could be more sensitive to uncontrolled environmental changes.

The anthocyanin content followed a second-degree pattern during the course of grain filling. Anthocyanins were synthesized relatively late in grain filling, reaching a peak at maturity and then decreasing through grain desiccation. This is in line with other findings for wheat [17] and rice [31]. This could be due to differences in the development rate between the endosperm and external layers [32]. The reduction after the maximum anthocyanin content occurred in the phase of desiccation, that is, when the grain filling period was almost completed and thus was not caused by a biomass dilution effect. On the contrary, Bustos et al. [33] suggested that the reduction of the anthocyanin content was conditioned by the availability of assimilates in the final phase of grain filling.

The anthocyanin content decreased in the stressed treatment. Imposing high temperatures by covering the field plots with a polyethylene film also reduced the incident radiation and this decrease could be likely the cause for the reduction in the anthocyanin content. The genes that control anthocyanin biosynthesis are positively regulated by light [34], so the incidence of solar radiation had a direct effect on the accumulation of anthocyanins. Bustos et al. [33] also observed a decrease in the anthocyanin content in wheat grains from shading the ears. Anthocyanins acted as a specific light protector and their high content favored the absorption and tolerance to ultraviolet radiation, as well as increasing their antioxidant capacity [35]. Therefore, blocking UV radiation by conventional polyethylene film [36] affected the rate of anthocyanin deposition in the barley grains.

The free phenolic compounds decreased toward grain maturation for all four genotypes in both stress and control conditions, as also reported in wheat during grain development [37]. The bound phenol concentrations did not follow this linear trend, rather they followed a second-degree curve as the grain developed. The bound phenols in Hindukusch, the purple grain genotype, increased sharply toward the end of grain filling. Ma et al. [16] reported that the highest bound phenol biosynthesis occurs later in the development of purple wheat than in the non-colored genotypes. In a previous study, we observed that the main phenols detected in the bound phenolic fraction in the mature grain were phenolic acids [24]. These compounds were also detected to a lesser extent in the free phenolic fraction. Furthermore, among these genotypes, Hindukusch had the highest bound phenolic acid content [24]. Therefore, the bound phenol accumulation could be attributed to conversion between fractions (from free to bound) during grain filling.

The higher levels of phenolic compounds in the early stages of grain filling may be a consequence of an early activity of phenylalanine ammonia lyase (PAL) [38], the enzyme that catalyzes the conversion of phenylalanine to trans-cinnamic acid during phenolic compounds biosynthesis [35]. The reduction in phenolic compounds during grain filling was mainly due to a dilution effect, as starch was deposited in the growing endosperm [17]. Several studies have speculated that sucrose increased PAL activity and induced the production of phenolic compounds during the development of different plant species [39,40,41]. The carbohydrate availability could have been compromised primarily during starch synthesis in later grain filling stages [9], affecting the phenolic compound biosynthesis during the final grain development. Other authors suggested that the reduction in phenolic contents during grain maturation was more closely related to other physiological processes, such as a decrease in photosynthesis or oxidative metabolism during the grain dehydration process [37].

Genotypic effects were more important than environmental conditions for free and bound phenolic contents. However, free and bound phenolic contents changed differentially between genotypes with the severity of heat stress. Our results show that the bound phenols were more stable under high temperature stress while the free phenols showed greater variability to thermal stress, corroborating the results observed by Silvestro et al. [42]. These authors showed that free phenols increased more under harsh climatic conditions than bound phenols. However, this was not observed for the colored genotype under stress. This showed less free phenol content than under the control conditions. The reduction of anthocyanins associated with the polyethylene film covering could explain why the concentrations of free phenols did not increase in this genotype. Heat stress also had a significant impact on the bound phenol content in all genotypes. An increased rate of bound phenol accumulation was sustained throughout the period of high temperatures in all genotypes. Such high temperatures have proven favorable to phenolic synthesis in wheat varieties [43]. Plants synthesized more phenolic compounds under temperature stress, which ultimately protected these plant cells from heat-induced oxidative damage [35]. Therefore, the increased accumulation of these compounds was accompanied by enhanced tolerance of the plants to high temperatures. Previous research suggested that high temperature influenced the metabolic pathway of phenolic compounds by augmenting the PAL activity, thus increasing the content of some phenolic compounds, which protected plants against heat stress [38,44].

The antioxidant properties constantly decreased during grain development, as reported in rice [31] and wheat [37]. Antioxidant molecules had less activity in the final stage of grain maturation due to the decrease in some physiological processes, such as photosynthesis or oxidative metabolism during the grain dehydration process, as proposed by Özkaya et al. [37]. Reactive oxygen species were generated during these physiological processes. Thus, if these processes were decreased during the grain dehydration (final stage of grain filling), the antioxidant molecules levels generated by the grain decreased consequently. Hence, the antioxidant capacity progressively decreases as the grain matures. Free and bound phenols make a significant contribution to the total antioxidant capacity, as described in the literature [7,45]. The increase in the antioxidant capacity due to heat stress was related to the increase in phenolic compounds. This could have occurred to protect the plant cells from heat-induced oxidative damage, as mentioned above.

## 4. Materials and Methods

### 4.1. Field Site Description, Treatments and Experimental Conditions

The field experiments were carried out under irrigated and well-fertilized conditions during the 2016–2017 and 2017–2018 growing seasons at Semillas Batlle SA, Bell-Lloc d’Urgell (41°37′ N, 0°47′ E), Northeast Spain. Four food barley genotypes were grown under two temperature conditions after heading. The genotypes were Annapurna, Hindukusch, Hispanic and Tamalpais, parents in our food barley breeding program, and they differed in number of rows, presence/absence of hulls, type of starch, grain quality and color (their grain characteristics appear in Table S1). The temperature treatments were: a control and a high temperature treatment starting 15 days after heading (decimal code [46], DC55) and continuing to physiological maturity (DC 90). The main plot size was 4 m × 1.5 m (six rows separated 20 cm) from which two subplots of the same size were established in order to apply the control and high temperature treatments from mid-April to late June. The seeding rate was 350 seeds/m^2^. All plots (control & heat stress) were flood-irrigated twice, once before heading and a second time in the first part of grain filling (@ 60 mm each time), to assure that they did not suffered of any water stress. Biotic interferences were avoided through controlling weeds, insects and diseases following usual practices. The heat treatment was imposed by covering half of the plot with transparent polyethylene film (125 μm) mounted on wooden structures 1.5 m above the soil level, as described in Elía et al. [25] but leaving the bottom 30 cm of the four sides of each structure open and the top punctured in order to facilitate free gas exchange. To monitor the air temperature, regularly distributed temperature sensors connected to data loggers were placed inside and outside the structures at the height of the spikes. The structures increased the maximum temperature by up to 8 °C, while the polyethylene film reduced solar radiation by up to 15%. The average daily temperatures in the spring growing period were higher (15 °C vs. 13 °C), precipitation lower (100 L/m^2^ vs. 175 L/m^2^) and solar radiation more intense (+10% vs. −10% long-term average) in 2017 than 2018 [47]. For more details see Martínez et al. [24].

### 4.2. Grain Weight and Milling

Individual spikes were marked at anthesis to monitor grain growth. At seven-day intervals during grain development, from seven days after anthesis until harvest maturity, ten spikes were collected from each subplot. The spikes (a total of seven samples per subplot) were lyophilized using a HALDRUP LT-15 laboratory thresher (HALDRUP GmbH, Ilshofen, Germany). Once the grains were threshed, thousand grain weight (GW) was determined with a Marvin system (GTA Sensorik GmbH, Neubrandenburg, Germany) according to the standard MSZ 6367/4-86 (1986) method. The grains were then milled using a Foss Cyclotec 1093™ (FOSS, Barcelona, Spain) mill equipped with a 0.5 mm screen. Finally, the flour was immediately stored at −20 °C in the dark until analysis.

### 4.3. Determination of β-Glucans and Arabinoxylans

Total β-glucan and arabinoxylan contents were measured, respectively, by the mixed-linkage β-glucan assay (K-BGLU) [48] and D-xylose assay (K-XYLOSE) [49] kits from Megazyme (Wicklow, Ireland), according to the manufacturer’s instructions.

### 4.4. Determination of Anthocyanins

Analysis of anthocyanins was carried out according to Abdel-Aal and Hucl [50]. The absorbance was read at 535 nm using a Multiscan GO spectrophotometer (Thermo Scientific, Vantaa, Finland). The anthocyanins were quantified with a standard calibration curve obtained for the Cyanidin-3-glucoside and expressed as µg Cy-3-glu/g.

### 4.5. Determination of Free and Bound Phenolic Compounds

Free and bound phenolic compounds were determined the second year, using the extraction method reported by Martínez et al. [8]. Both fractions were then analyzed using the spectrophotometric Folin−Ciocalteu method adapted to a microplate format by Bobo-García et al. [51]. The absorbance was read at 760 nm using a Multiscan GO spectrophotometer (Thermo Scientific, Vantaa, Finland). The phenolic compounds were quantified with a standard calibration curve obtained for the Gallic acid equivalent (GAE) and expressed as mg GAE/g.

### 4.6. Determination of Antioxidant Capacity

Antioxidant capacity (AC) was also measured in the second year with the oxygen radical absorbance capacity (ORAC) assay, according to Huang et al. [52]. Trolox (6-hydroxy-2,5,7,8-tetramethylchroman-2-carboxylic acid) was used as the control. Antioxidant capacity was expressed as μmols Trolox equivalent/g.

### 4.7. Statistical Analysis

All bioactive compound contents were referred to grain weight, either as a mg/g, µg/g and µmols/g depending on their final concentration. In order to characterize the deposition profile of the different bioactive compounds, the Specialized Modelling procedure in JMP14 Pro (SAS institute Inc., Cary, NC, USA) was used to fit the average of two blocks for each genotype × year × environment combinations simultaneously, using growing degree-days after anthesis (GDA), determined using maximum and minimum daily temperature with a based temperature of 0 °C, as thermal time. Nonlinear standard 3-parameter logistic curve and alternative polynomial linear models were compared. The best model was identified using the minimum value of the Akaike information criterion. JMP14 directly provides the estimates and standard errors for the 3-parameters of the logistic sigmoid for each curve: growth rate, inflection point and maximum value. As the 3-parameter logistic curve is symmetrical around its inflection point, we approximated duration and its standard error by multiplying the inflection point and its standard error by 1.9. In other words, we defined duration as the thermal time needed to increase the content from 5% to 95% of the final value. In order to study the dynamics of the contents of the different bioactive compounds during the course of grain filling, whenever the logistic model was selected, we compared the estimates for the three parameters determined for each of the curves directly by means of simple analysis of variance. As the use of standard errors as weights did not alter the results, simpler unweighted ANOVA models were preferred. The number of curves did not allow for a full three-factor factorial expansion. Therefore, two- and three-way interactions, with the exception of the fixed genotype × environments (i.e., stressed vs. control conditions) interaction, were pooled into a single error term. Deposition patterns for bioactive compounds following a linear polynomial model were directly studied by incorporating first- or higher-order thermal time terms into a standard covariance linear model.

## 5. Conclusions

Barley cultivated in a heat-stressed area, specifically under Mediterranean conditions, is a valuable source of dietary fiber, phenolic compounds and antioxidant capacity for cereal-based healthy food products. In general, the contents of the bioactive compounds were determined more by the genotype than by the environment. Induced late high-temperature stress reduced final grain weight, did not affect the β-glucans or arabinoxylan contents and increased the phenolic compounds, as well as their antioxidant capacity, especially for Annapurna and Hispanic. However, future research would be necessary to determine whether the structure of some of these bioactive compounds is affected during grain accumulation in high-temperature stress-prone areas, as it can influence the quality of the barley-based products. The concentration of bioactive compounds changed differentially throughout grain filling, depending on the development time when they were synthesized. The deposition patterns for dietary fiber followed that of grain weight. Annapurna needed less thermal time to increase the grain weight and β-glucans content from 5% to 95% of the final value during grain filling. Anthocyanins reached a maximum before the end of grain filling. The rate of anthocyanins deposition changed under control and stress conditions, likely due to the plastic covering used to increase the temperature that reduced the solar radiation, decreasing the anthocyanin content in the colored genotype. Free and bound phenols constantly decreased as the grains developed, suggesting that they are synthesized in early stages. However, it was observed that the rate of deposition of bound phenols was more sensitive to genotypic changes; Hindukusch, the purple genotype, behaved differently than the other genotypes. Overall, the antioxidant capacity decreased over time, but the decay observed in the control plots was steeper than for the stress-induced ones. These results support food barley cultivation in high-temperature stress-prone areas, as some bioactive compound and antioxidant capacity will increase, regardless of the smaller size grains. Furthermore, if a market develops for food barley ingredients, early harvesting of non-mature grains should be considered to maximize antioxidant capacity.

## Figures and Tables

**Table 1:** Analyses of Variance for the Estimates of the Grain Weight (mg) Deposition Parameters: Maximum Weight, Growth Rate and Duration.

Source	df	Maximum Weight			Growth Rate			Duration		
		Sum of Squares	*F* Ratio	*p*-Value	Sum of Squares	*F* Ratio	*p*-Value	Sum of Squares	*F* Ratio	*p*-Value
Corrected total	15	582.0			0.000028960			87,893		
Year	1	64.1	4.78	*0.0651*	0.000000004	0.00	*0.9887*	44,123	28.62	***0.0011***
Genotype: G	3	188.0	4.67	***0.0427***	0.000001001	1.74	*0.2579*	25,485	4.95	***0.0376***
Environment: E	1	191.3	14.26	***0.0069***	0.000006260	3.27	*0.1207*	3.000	0.00	*0.9699*
G × E	3	44.3	1.11	*0.4061*	0.000003110	0.54	*0.6745*	1286	0.25	*0.8591*
Residual	7	93.9			0.000011500			12,016		

A bold number indicates statistical significance at α < 0.05.

**Table 2:** Analyses of Variance for the Estimates of β-glucan Content (mg/g) Deposition Parameters: Maximum Content, Growth Rate and Duration.

Source	df	Maximum Content			Growth Rate			Duration		
		Sum of Squares	*F* Ratio	*p*-Value	Sum of Squares	*F* Ratio	*p*-Value	Sum of Squares	*F* Ratio	*p*-Value
Corrected total	15	3112.0			0.00017606			94,632		
Year	1	2.2	0.10	*0.7571*	0.00000210	0.22	*0.6573*	4340	1.22	*0.3065*
Genotype: G	3	2946.9	46.05	***0.0001***	0.00007037	2.39	*0.1543*	51,292	4.79	***0.0403***
Environment: E	1	11.8	0.55	*0.4830*	0.00000166	0.17	*0.6933*	5412	1.52	*0.2578*
G × E	3	2.4	0.04	*0.9885*	0.00003325	1.13	*0.4003*	8617	0.81	*0.5297*
Residual	7	148.7			0.00006867			24,971		

A bold number indicates statistical significance at α < 0.05.

**Table 3:** Analyses of Variance for the Estimates of Arabinoxylan Content (mg/g) Deposition Parameters: Maximum Content, Growth Rate and Duration.

Source	df	Maximum Content			Growth Rate			Duration		
		Sum of Squares	*F* Ratio	*p*-Value	Sum of Squares	*F* Ratio	*p*-Value	Sum of Squares	*F* Ratio	*p*-Value
Corrected total	15	674.1			0.00016401			523,370		
Year	1	165.8	4.51	*0.0714*	0.00002439	2.25	*0.1775*	227,143	9.03	***0.0198***
Genotype: G	3	233.3	2.11	*0.1870*	0.00005678	1.75	*0.2436*	102,788	1.36	*0.3304*
Environment: E	1	16.0	0.43	*0.5310*	0.00000077	0.07	*0.7973*	7346	1.29	*0.6057*
G × E	3	1.3	0.01	*0.9981*	0.00000652	0.20	*0.8925*	9969	0.13	*0.9379*
Residual	7	257.6			0.00007566			176,124		

A bold number indicates statistical significance at α < 0.05.

**Table 4:** Analysis of variance for anthocyanin content (µg Cy-3-glu/g) according to a second-degree curve for thermal time.

Source	df	Sum of Squares	*F* Ratio	*p*-Value
Corrected total	27	343,845		
Thermal time: TT	2	187,749	32.24	***0.0000***
Year: Y	1	3215	1.10	*0.3090*
Environment: E	1	49,574	17.03	***0.0008***
Y × E	1	3111	1.07	*0.3166*
TT × Y	2	12,908	2.22	*0.1413*
TT × E	2	37,208	6.39	***0.0091***
TT × Y × E	2	3498	0.60	*0.5603*
Residual	16	46,581		

A bold number indicates statistical significance at α < 0.05.

**Table 5:** Analyses of variance of total free and bound phenolic compound contents (mg Gallic acid equivalent (GAE)/g) and antioxidant capacity (µmol Trolox/g) according to a first- or second-degree curve with thermal time.

Source	Free Phenols (First-Degree Model)				Bound Phenols (Second-Degree Model)				Antioxidant Capacity (First-Degree-Model)			
	df	Sum of Squares	*F* Ratio	*p*-Value	df	Sum of Squares	*F* Ratio	*p*-Value	df	Sum of Squares	*F* Ratio	*p*-Value
Corrected total	55	7.751			47	11.95			55	46,131		
Thermal time: TT	1	3.364	92.31	***0.0000***	2	0.75	22.78	***0.0000***	1	29,490	390.43	***0.0001***
Genotype: G	3	1.392	12.73	***0.0000***	3	6.44	129.70	***0.0000***	3	15,590	68.80	***0.0001***
Environment: E	1	0.409	11.22	***0.0027***	1	2.80	169.11	***0.0000***	1	3274	43.34	***0.0001***
G × E	3	0.793	7.25	***0.0013***	3	0.08	1.66	*0.2030*	3	1073	4.73	***0.0064***
TT × G	3	0.142	1.30	*0.2981*	6	1.42	14.26	***0.0000***	3	125	0.55	*0.6495*
TT × E	1	0.127	3.48	*0.0743*	2	0.01	0.33	*0.7206*	1	909	12.03	***0.0013***
TT × G × E	3	0.068	0.62	*0.6088*	6	0.06	0.61	*0.7226*	3	190	0.84	*0.4801*
Residual	40	1.458			24	0.40			40	3021		

A bold number indicates statistical significance at α < 0.05.

## Supplementary Materials

The following are available online at https://www.mdpi.com/2223-7747/10/3/598/s1, Table S1: Grain characteristics and average final content of grain weight (GW), β-glucans, arabinoxylans, total phenolic compounds and antioxidant capacity (AC) of four barley genotypes during two consecutive seasons of the control samples without heat stress.

## Abbreviations

AC: Antioxidant Capacity; Cy-3-glu: Cyanidin-3-glucoside; DC: Decimal Code; GAE: Gallic Acid Equivalent; GDA: Growing Degree-days accumulated after Anthesis; GW: Grain Weight; ORAC: Oxygen Radical Absorbance Capacity; PAL: Phenylalanine Ammonia Lyase.
