# Supplementary material for: Impact of Rising Temperature in the Deposition Patterns of Bioactive Compounds in Field Grown Food Barley Grains
Source: Plants (Basel). 2021 Mar 22;10(3):598. doi: 10.3390/plants10030598 (PMC8004944; doi:10.3390/plants10030598)
Supplement: Supplementary file 1 [file plants-10-00598-s001.pdf]

plants

MDPI

Supplementary Files

Impact of rising temperature in the deposition patterns of bioactive compounds in field grown food barley grains

Mariona Martínez-Subirà, Marian Moralejo, Eva Puig, MariPaz Romero, Roxana Savin, Ignacio Romagosa*.

University of Lleida, AGROTECNIO-CERCA Center, Av. Rovira Roure 191, 25198 Lleida, Spain
*Correspondence: ignacio.romagosa@udl.cat; Tel.: +34 973 702534

Table S1. Grain characteristics and average final contents of grain weight (GW), β-glucans, arabinoxylans, total phenolic compounds and antioxidant capacity (AC) of four barley genotypes during two consecutive seasons of the control samples without heat stress.

Genotype		Endosperm	Hull	Row	Colour	β-glucan content	Average final contents of two growing seasons GW (mg)	β-glucan (mg/ g)	Arabinoxylan (mg/ g)	Phenolic compounds (mg/ GAE g)	AC (µmolsTrolox/ g)
Annapurna	Spanish variety	Waxy	Naked	2	Yellow	High	51 a	79 a	55 a	4.6 bc	111 b
Hindukusch	Afghan landrace	Non-waxy	Partially naked	2	Purple	Medium	47 ab	68 b	55 a	5.7 a	140 a
Hispanic	Spanish variety	Waxy	Hulled	2	Yellow	Low	52 a	50 c	41 c	4.2 c	91 c
Tamalpais	North American variety	Waxy	Naked	6	Yellow	High	44 b	80 a	45 b	4.9 b	110 a

Results are presented as mean. Mean with a column followed by different letters indicate significant differences; Tukey-Kramer ($\alpha = 0.05$).
